# Supplementary material for: First of a line or last of a dynasty? Parabos tigneresi and the evolution of eurasian bovinae in the early pliocene
Source: PLoS One. 2026 Jun 3;21(6):e0340256. doi: 10.1371/journal.pone.0340256 (PMC13232859; doi:10.1371/journal.pone.0340256)
Supplement: Attachment — Submitted filename: Response to Reviewers.docx [file pone.0340256.s040.docx]

**AUTHORS’ RESPONSE TO THE EDITOR AND REVIEWER’S COMMENTS**

**Journal:**	PLOS One

**Manuscript #:**	PONE-D-25-47590R1

**Title of Paper:**	First of a Line or Last of a Dynasty? Parabos tigneresi and the Evolution of Eurasian Bovinae in the Early Pliocene

**Authors:**	Leonardo Sorbelli, Faysal Bibi, Joan Madurell-Malapeira, Federica Grandi, Elena Moreno-Ribas, Oriol Oms, Gerard Campeny, Bruno Gómez de Soler

**Dear Editor and Reviewer Dimitris S. Kostopoulos,**

We thank you and reviewer D. S. Kostopoulos for the thorough evaluation of our submitted manuscript. We appreciate that the paper has been well received and are grateful for the reviewer’s particularly kind and constructive comments.

We have addressed all the issues indicated in the review report and we made very few minor changes to the original text. We also provide new versions of the Figures 1, 2, 17, 18, 19, 20 modified according to the requests of the editor and reviewer.

Included with this resubmission are the three required files: (1) a marked-up copy of the revised manuscript (“Revised Manuscript with Track Changes”), (2) a clean version of the revised manuscript (“Manuscript”), and (3) the rebuttal document containing our detailed responses to reviewer’s comments (“Response to Reviewers”). As shown in both the rebuttal and the marked-up manuscript, all changes suggested by the reviewer have been fully implemented.

As shown in both this document and the marked-up manuscript, all changes suggested by the reviewer have been fully implemented.

We believe that the revised version can meet the journal publication requirements.

Below, we address each point raised by the editor in the journal’s requirements section.

1. **Please ensure that your manuscript meets PLOS ONE's style requirements […]**

Completed.

2. **In your manuscript, please provide additional information regarding the specimens used in your study […]**

No additional information was required.

In the acknowledgments we added the phrase: “No permits were required for the described study, which complied with all relevant regulations.”.

If this sentence should be placed elsewhere in the manuscript, please let us know.

3. **Thank you for stating the following financial disclosure […]**

The founders had no role in study design, data collection and analysis, decision to publish, or preparation of the manuscript.

4. **Thank you for stating the following in the Acknowledgments Section of your manuscript […]**

The funding-related text has been removed from the acknowledgments section.

Please implement the following amended version of the funding acknowledgments:

“The CN project is supported by the Caldes de Malavella Town Hall. This research has received funding from the Catalan Government (Generalitat de Catalunya) through the Departament de Cultura (project CLT009/22/000043) and the research group 2021 SGR 01238 (AGAUR), as well as from the Universitat Rovira i Virgili (grant 2023-URV-01238). Additional funding is provided by the Ministry of Science and Innovation of the Spanish Government through project PID2024-157622NB-I00. L.S. was supported by a Humboldt Research Fellowship provided by the Alexander von Humboldt Foundation and by the CERCA Programme of the Generalitat de Catalunya. The research conducted by F.G., E.M., G.C., and B.G.S. is funded by the CERCA Programme of the Generalitat de Catalunya. The IPHES-CERCA has received financial support through the “María de Maeztu” program for Units of Excellence (CEX2024-01485-M/funded by MICIU/AEI/10.13039/501100011033).”.

5. **We note that Figure 1 and 2 in your submission contain [map/satellite] images which may be copyrighted […]**

Both figures 1 and 2 have been modified accordingly. The map in Figure 1 has been redrawn by us using original data in our possession, and the map in Figure 2 has been produced using data from one of the recommended platforms (http://www.naturalearthdata.com/)

6. **Please include captions for your Supporting Information files at the end of your manuscript […]**

Completed.

7. **If the reviewer comments include a recommendation to cite specific previously published works […]**

No suggested additional references were given by the reviewer

8. **Please review your reference list to ensure that it is complete and correct […]**

The reference list was checked to ensure that is correct. We note one issue regarding the reference for the new dating of the Camp dels Ninots site. The study establishing its Early Pliocene age is still under review. For the moment, we have kept the citation as “(in prep.)”. We are unsure when it will be published, but the results supporting the age are robust and consistent with the site’s biochronological framework. Please advise if an alternative approach is required.

Below, we address each point raised by the Reviewer.

**Response to general comments:**

**Reviewer’s comment:** “A truly excellent and important piece of work that provides valuable information about the relatively unknown Bovinae of the Early Pliocene in Europe based on the impressive material from Camp dels Ninots. The descriptions are detailed and accurate, the comparisons comprehensive and thorough, the diagrams and visual material impeccable, as usual in works signed by the first author, the bibliography is fully up to date, and the conclusions are interesting and in complete harmony with the results. I have very few comments/corrections and suggestions to make (see annotated pdf). Some of the environmental/biozoogeographical interpretations may be somewhat exaggerated (see comments in pdf) and would be better formulated as working hypotheses for future studies.”

**Answer:** we thank the reviewer for the generous comments regarding our work. We appreciate that this research has been well received, and we hope it will improve future discussions about the evolution of Bovinae in Europe. We changed the text and figures according to the suggestions and corrections given by the reviewer.

**Response to specific comments:**

Line numbers cited in this letter refer to the original version of the manuscript.

**Page 1, line 35.** Reviewer’s comment: “P. ....Parabos tingeresi I suppose”

Answer: typo corrected.

**Page 2, line 59.** Reviewer’s comment: “assemblage”

Answer: suggestion accepted.

**Page 2, line 59.** Reviewer’s comment: “ ”

Answer: space added.

**Page 2, line 66.** Reviewer’s comment: “since Zanglean, Piacenzian...are Marine stages they sound peculiar when used for continental deposits”

Answer: changed Zanclean to Early Pliocene and Piacenzian to Late Pliocene in the whole text.

**Page 2, lines 69-70.** Reviewer’s comment: “Is that age fixed? is the article accepted?”

Answer: The article is currently under review therefore we do not know, at the moment, when it will be published. However the results for the age are extremely robust and agreeing with the biochronological framework of the site.

**Page 3, line 77.** Reviewer’s comment: “a large bovid referred to as....(becasue you later use a different combination)”

Answer: suggestion accepted.

**Page 3, lines 81-82.** Reviewer’s comment: “I think it is not anymore necessary if you accept my previous addition”

Answer: Deleted as suggested.

**Page 3, line 101.** Reviewer’s comment: “black letterin?”

Answer: comment unclear.

**Page 4, line 114.** Reviewer’s comment: word “measurable” deleted

Answer: suggestion accepted.

**Page 5, line 149.** Reviewer’s comment: “taken at +++++”

Answer: clarified in the text as: “taken at its highest point”.

**Page 5, line 149.** Reviewer’s comment: “occlusal/basal?”

Answer: clarified in the text as: “basal”.

**Page 7, lines 230-231.** Reviewer’s comment: “you mean parallel? becasue emerging upright calls for their position relative to the orbits......”

Answer: Modified according to the suggestion as “emerging parallel or slightly diverging”.

**Page 8, line 268.** Reviewer’s comment: “new combination (may be useful to be added)”

Answer: “comb. nov.” added to the text.

**Page 10, line 306.** Reviewer’s comment: “again this expression is a bit confusing...”

Answer: “upright but” deleted.

**Page 10, line 322.** Reviewer’s comment: “length”

Answer: added in the text as suggested.

**Page 10, line 336.** Reviewer’s comment: “but you-correctly-state the braincase is short...”

Answer: Modified in the text as: “relatively short”.

**Page 11, line 341.** Reviewer’s comment: “short and high?”

Answer: By “shallow temporal fossa” we mean that the fossae do not deepen into the neurocranium; the temporal crests do not overhang the fossae, unlike in more derived members of the Bovini such as *Leptobos*. Clarified in the text as: “The temporal fossae are shallow (i.e., the parietal crests do not overhang the fossae) and high”.

**Page 11, line 345.** Reviewer’s comment: “OR equally expanded bith laterally and posteriorly?”

Answer: Clarified in the text as: “The mastoid area is enlarged, greatly expanded laterally and posteriorly.”.

**Page 11, line 371.** Reviewer’s comment: “ir” deleted in “Their”

Answer: typo corrected.

**Page 14, line 431.** Reviewer’s comment: “I am not sure what do you mean here - a goat fold usually refers to the lower molars”

Answer: sentence deleted.

**Page 16, line 487.** Reviewer’s comment: word “with” deleted.

Answer: typo corrected

**Page 18, lines 515-516.** Reviewer’s comment: “as in all bovids,isn't it? so better to avoid banalities”

Answer: deleted as suggested.

**Page 18, line 518.** Reviewer’s comment: “Yes for LM but not for P Q in Fig 12....why? ont age? sex?”

Answer: we do not know the reason of this difference with certainty bu it is possible that it is related to sex. Both individuals are old but the stoutest might be male. Clarified in the text as: “It possesses a relatively thin diaphysis in the most gracile specimens and more robust shaft in the largest individuals (e.g., IPHES.CN'04-B1-303 and IPHES.CN'11-B4-303; Fig 12J–Q; S9 Table).”.

**Page 18, line 520.** Reviewer’s comment: “sure about that?”

Answer: “strong” deleted to comply with the suggestion.

**Page 18, lines 523-524.** Reviewer’s comment: “banalities”

Answer: text shortened but the difference in size of the two facets was left because it varies within Bovinae.

**Page 18, line 35.** Reviewer’s comment: “again in Fig 13, M,N & U,V less wide than Q,R”

Answer: added a phrase referring to this difference: “Its robusticity changes accordingly to the individual (e.g., slenderer in IPHES.CN'19-B15-432 and stouter in IPHES.CN'11-B4-306).”

**Page 18, line 526.** Reviewer’s comment: word “greatly” deleted.

Answer: suggestion accepted.

**Page 19, line 554.** Reviewer’s comment: “banality”

Answer: phrase cancelled as suggested.

**Page 19, line 558.** Reviewer’s comment: “why short if it has the same length as the femur? short compare to whet?”

Answer: text modified as: “The tibia is relatively stout, roughly of the same length of the femur”.

**Page 19, lines 561-564.** Reviewer’s comment: “again, try to avoid some banalities typical of Bovidae/Bovinae”

Answer: text shortened as suggested.

**Page 20, lines 565-565.** Reviewer’s comment: “same”

Answer: text shortened as suggested.

**Page 20, line 588.** Reviewer’s comment: “same..and several others in between....”

Answer: Text from line 565 to 600 shortened in various points according to the suggestion.

**Page 20, line 590.** Reviewer’s comment: “compared to what - it seems normal to me”

Answer: Modified as: “The intermediate phalanx is shorter”.

**Page 20, lines 597-598.** Reviewer’s comment: “isn't this the normal condition?”

Answer: actually this condition is stronger in some bovids that prefers a certain kind of substrate (see discussions, pag. 40).

**Page 22, line 642.** Reviewer’s comment: “Extant ”

Answer: suggestion accepted.

**Page 22, line 662.** Reviewer’s comment: “is it?”

Answer: yes, in Alephis this torsion is more evident although not extreme in general terms.

**Page 22, line 665.** Reviewer’s comment: “postero-”

Answer: added in the text as suggested.

**Page 23, line 679.** Reviewer’s comment: “please note that in Tragoportax the posterointernal angle is well marked as well (not a true keel but a 'dihedre') as in several Parabos samples”

Answer: the reviewer is right. Modified in the text as: “the overall smaller size, a stronger mediolateral compression and, often (as it is the case of *Miotragocerus*), only two well-defined keels”.

**Page 23, line 703.** Reviewer’s comment: “Correct Samokoerus to Samokeros in most figs legends”

Answer: typo corrected the interested figures.

**Page 24, line 727.** Reviewer’s comment: “colors on legend bars to match is possible better colors on box plots”

Answer: box plots and legend bars are already matching at best.

**Page 26, line 767.** Reviewer’s comment: “17”

Answer: typo corrected.

**Page 26, line 767.** Reviewer’s comment: “D”

Answer: missing letter added.

**Page 26, line 767.** Reviewer’s comment: “, 20”

Answer: missing number added.

**Page 26, line 769.** Reviewer’s comment: “18D”

Answer: typo corrected.

**Page 27, line 799.** Reviewer’s comment: “rests ”

Answer: modified as suggested.

**Page 27, line 799.** Reviewer’s comment: “with wear”

Answer: added as suggested.

**Page 32, line 898.** Reviewer’s comment: “a or e?”

Answer: Tha Chang is correct, it is the name of the site.

**Page 35, line 1018.** Reviewer’s comment: “italics”

Answer: font modified.

**Page 35, line 1026.** Reviewer’s comment: word “that” deleted

Answer: repetition deleted.

**Page 36, line 1047.** Reviewer’s comment: “on ???”

Answer: error corrected in: “taxon”.

**Page 36, line 1064.** Reviewer’s comment: “LGPUT

Milia collection takes part of the LGPUT ”

Answer: error corrected.

**Page 37, lines 1084-1085.** Reviewer’s comment: word “derived ” moved in the text

Answer: suggestion accepted.

**Page 39, line 1164.** Reviewer’s comment: “oscillation?”

Answer: suggestion accepted.

**Page 39, line 1169.** Reviewer’s comment: “,”

Answer: suggestion accepted.

**Page 39, line 1169.** Reviewer’s comment: word “and” deleted.

Answer: suggestion accepted.

**Page 39, lines 1172-1178** Reviewer’s comment: “I am not really convinced....if early Bovini tend to adapted in drier climate and vegetation and already present in C Asia and E Africa at late Miocene, why not invaded then in Europe (at least in the East) instead of the humid phase of early Pliocene...it makes no real sense to me.... On the other hand Parabos does not seem to have a similar counterpart in Asian faunas.... Just thoughts for thoughts.....”

Answer: Reformulated in a less clear-cut way following the rightful critic of the reviewer as: “The westward spreading of C_4_ grass during the later Miocene led to the expansion of these early Bovini in the Middle East and Africa [69]. In contrast, the first bovin-like dentitions in Western Europe only appear in the Pliocene, with *Parabos* and *Alephis*. Whether the more humid and forested habitats of Western Europe [106, 113] were responsible for the delay in the appearance of these traits in the continent is still to be demonstrated. Crown Bovini with fully developed bovin dentition appears in Africa and South Asia by the earliest Pliocene (e.g., *Ugandax coryndonae, Proamphibos* spp.) while in Europe this is only with the arrival of the first *Leptobos* from the Eastern Palaearctic at the very end of Pliocene [22, 28 and reference therein]”.

**Page 40, line 1217.** Reviewer’s comment: “ ”

Answer: space added.

**Page 41, line 1229.** Reviewer’s comment: “s” deleted

Answer: typo corrected.

**Page 41, line 1229.** Reviewer’s comment: “ó”

Answer: comment unclear, possible error of the reviewer?

**Page 42, line 1268.** Reviewer’s comment: word “and” deleted.

Answer: suggestion accepted.

**Page 42, line 1268.** Reviewer’s comment: “,”

Answer: suggestion accepted.

**Page 1, line 35.** Reviewer’s comment: “If you wish, you could also mention the Museum of Geology, Paleontology, and Paleoanthropology (LGPUT) here.”

Answer: institutional acknowledgements section expanded as suggested.

Sincerely,
**Leonardo Sorbelli**
on behalf of all co-authors
